# Lectures on Clinical Medicine, Delivered at the Philadelphia Medical Institute

**Published:** 1838-05-23

**Authors:** W. W. Gerhard

**Affiliations:** Physician to the Philadelphia Hospital, &c.


### CLINICAL LECTURES.

LECTURES ON CLINICAL MEDICINE, *delivered at the Philadelphia Medical Institute, by* W. W. GERHARD, M. D., *Physician to the Philadelphia Hospital,* &c.

### INFLAMMATIONS OF SEROUS MEMBRANES.

*Tuesday, May* 1*st.* In my lectures on pathological anatomy, I have already pointed out to you the distinctive characters of the inflammations of the serous membranes. As you, no doubt, remember, these characters consist in the bright arterial injection of the membrane, and the secretion into its cavity of lymph, serum, and pus. The lymph is secreted very early; I once detected it in a case of pneumothorax which proved fatal in an hour after the perforation: at first it is secreted in the form of minute points scattered thickly over the inflamed surface; as these points become more numerous, they gradually run together, until the whole surface is covered by a tolerably uniform coating of lymph, which is then called a false membrane. Portions of the lymph are afterwards detected in flakes interspersed through the serum. The liquid consists chiefly of serum which is slightly turbid from the admixture of lymph, and of a small portion of pus, which gives it a yellowish tinge. The pus is not abundant in acute inflammations, and in subjects of a feeble constitution is scarcely perceptible, but when the inflammation becomes chronic, the proportion of pus gradually becomes more considerable, until it at last forms the whole of the liquid. It is then called an empyema.

The manner in which the gradual absorption of the liquid occurs, and the two surfaces of lymph become organized, and finally adhere together, must also have become so familiar to you as to require no further description at present.

Serous inflammations may be either primary or secondary. When secondary, they are usually dependant, first, on the previous existence of inflammatory rheumatism, secondly, on disease of the organs which they invest, and thirdly, on the existence of a tuberculous diathesis. I shall not now dwell upon the rheumatic causes of serous inflammation, as I entered into this subject, at a former lecture, when speaking of the case of acute articular rheumatism in which pericarditis occurred. Its connection, too, with disease of the parenchymatous tissues covered by serous membranes, we shall discuss at length, on some future occasion, when we may see, that inflammation of the lungs, liver, that in fact, every lesion of an organ, gives rise to more or less inflammation of its serous covering. This process is an effort of nature to isolate and protect the diseased organ. The third cause of serous inflammation, that is, the existence of a tubercular diathesis, is the most complicated, and presents the most numerous points for examination: into some of these we shall enter this afternoon. The connection between serous inflammation and tubercles is the more important, from its enabling us to recognise a number of tubercular cases, otherwise obscure, by reasoning upon the law of pathology, that tubercles are so often connected with inflammation of these membranes.

In addition to those above mentioned, there may be other causes of secondary serous inflammation; they may be mechanical, as perforations, outward violence by blows, and the like; the influence of cold which may be felt in the membrane surrounding the joints, intestines, lungs and heart, produces a primary inflammation.

That secondary serous inflammations are not of much more frequent occurrence than they are, is to me a matter of surprise, when I reflect upon the close connection of the organs with their serous investment; they are comparatively of more importance, than the primary forms, from their more frequent fatality.

There are certain signs common to serous and other inflammations, by which they are generally ushered in. These are chill, fever, and sweating, with a general malaise, or feeling of wretchedness, loss of appetite, thirst, and in fact the ordinary febrile symptoms. They may, however, be entirely absent, and I have seen patients die without either fever, thirst, or severe pain. The pain accompanying serous inflammation is usually sufficiently indicative of its character. It may be said to be specific, being lancinating, sharp, acute, resembling such as would be inflicted by the stab of a knife. It is ordinarily described by patients in these terms; it may, however, be so slight as not to confine the patient to bed, in fact, not severe enough to enable him to localize the disease. Patients often come into the ward, with the general febrile symptoms, above mentioned, without local suffering enough to allow them to fix the particular seat of their disease. It is obvious, then, that we cannot trust to this character of pain, in determining the nature of the affection, but must resort to physical examination, the phenomena of which, resulting from the identity of the liquid secreted, will be always the same.

There will be besides a disordered action of the organ invested by the serous membrane; in pericarditis, of the heart, in pleurisy, of the lungs, and in peritonitis, although not so distinctly marked as in other inflammations, there is generally sufficient evidence of disturbance in the action of the bowels.

The state of the pulse is another sign, supposed to be specially characteristic of this disease. But you recollect that, in the case of the man Robb, the pulse was merely moderately frequent, seventy-six per minute, notwithstanding the pericardium was inflamed; and, in another patient, laboring also under intense serous inflammation, you noticed also that it was very nearly of the same frequency, that is, decidedly not febrile. The character of the pulse is, therefore, a faithless sign of the existence of serous inflammation; it may be peculiar, small, wiry, and of intense activity, in which case bleeding is demanded without delay; but these dictinctive features are not generally present.

In the patient noticed to-day, the pleurisy was nearly, though not entirely latent. The woman, whose case has been before you, had neither pain nor fever, although there was an effusion into the pleura of a quart and more in amount. The signs of this effusion were merely physical. This latent pleurisy is a common affection with old people, many of whom perish from it, when supposed to die simply from the decay of old age. In young persons these diseases are not so often latent, except in a very chronic form, or where the patient has been exhausted by chronic illness. An example of this was furnished by the case, that terminated yesterday fatally at the hospital. It occurred in a patient, who had been laboring under chronic peritonitis for a year previously, and who was taken about a week since with a slight increase of his ordinary pain, accompanied by severe prostration, which carried him off in a few days. An exception to this rule, however, occurred at the Pennsylvania Hospital three years since, in the case of a man, who was run over in the belly by a cart. He suffered but little pain, from the first day, and was afterwards suddenly carried off, although the only alarming symptom was extreme exhaustion. Very extensive peritonitis' existed, but without pain, although the intestines were covered with lymph. You see, therefore, how difficult it is to recognise the presence of serous inflammations, from the functional signs which are presented. If we had no other means of examination, or if we omitted physical exploration, because there were no special symptoms to arrest and direct our attention, we should be constantly in error. For example, it is a very common occurrence with labouring men, suffering from chronic effusions into the pleuræ, to complain of pain, not in the region of the pleura, but lower down, in the flanks, whence they are sometimes treated merely for lumbago. In such cases, there is absolutely no rational symptom to indicate the nature of the disease and the proper mode of treatment; it is by the local signs only, that the true character of the affection can be traced.

The duration of serous inflammations is by no means fixed. It may be divided into two periods, the one including the time that passes during the increase of the effusion; the other, that during its stationary and declining stage. After effusion has taken place, it is not possible to cure the patient abruptly; time is absolutely required for the consolidation of the false membrane, and for the absorption of the pus and serum that have been secreted. In all acute inflammations of serous membranes, if you see the case only after effusion has taken place, you may safely predict that your patient will not be well, at least, for several days; and the rapidity of the recovery will depend upon the quantity of liquid which has been effused, and the time it has remained in the pleura. But, if you are called to a case, say of pleurisy, at the beginning of the attack, while there is merely slight inflammation without much effusion, the patient may get well abruptly, and the morbid secretion may be limited to a little lymph, which is a necessary consequence of all serous inflammations.

I do not intend now to enter at length into the peculiarities of treatment, nor into a detail of the physical signs, belonging to the varieties of serous inflammations, except so far as they may be exemplified by the cases, which I shall bring before your notice. The remarks which I have just now made, were necessary in order to make clear a number of cases, the histories of which I shall presently relate to you. You must bear in mind the distinction, which I made between primary and secondary serous inflammations. The first of these is scarcely ever fatal; by interference, you may shorten its course, but you may expect it to get well under any circumstances. Inflammations of this character depend merely on atmospheric vicissitudes or other such cause, and are not preceded by a tubercular lesion, or connected with this diathesis, nor are they the result of a perforation, which is generally irremediable. When you have a secondary serous inflammation, you are to look upon it as an effort of nature for the preservation of the part; and, when, it is complicated with inflammation of the parenchyma of the organ which it invests, it is a curative and preservative process, and is not to be interfered with, unless it is of that severe character which endangers the life of the patient.

The first illustration, which I shall bring before you to-day, is the termination of the case of the man Robb. At the last lecture, I mentioned to you, that the rheumatism was almost cured. The affection of the heart was then, and is still, persisting, although it is now chiefly limited to the internal membrane of the heart, and the symptoms are still so severe, as to prevent the man's yet leaving his bed—a proof of the difficulty of suddenly arresting diseases of this nature. These cases of serous inflammation, occurring during an attack of rheumatism, generally assume a character altogether independent of the course of the latter affection: the great fire goes out, but the smaller burns slowly on. Not only, indeed, does this cardiac inflammation run its course, but it often leaves behind it organic changes in the heart, that may last for a succession of years or for life, in the form of thickening of the valves, or adhesion of the laminæ of the pericardium. Numerous cases of extensive disease of the heart take their origin in an attack of rheumatism; they may, on the other hand, be of a slight character, and entail no greater disturbance of the economy than slight palpitations, and an inability to use the same amount of active exercise as in perfect health.

The next case I shall notice is somewhat curious; it is pleurisy, occurring in a young Irishwoman, Isabella M'Gargee. In December, 1837, she was exposed to great fatigue, and suffered much mental anxiety during the illness of a relative; she was then taken ill with severe pain in the region of the heart, dyspnœa and palpitation; for these symptoms she was bled and blistered with relief. Her health, however, was not entirely restored; there was still palpitation, at times cough and some oppression. At the beginning of April, she worked very hard in a damp cellar, washing clothes, and was seized, in a day or two afterwards, with fever and pain in the right side, extending from the breast towards the nipple, and much increased by respiration, or by the cough, which was short and dry, not frequent. There was also pain caused by lying on the affected side, with considerable oppression. The gradual increase of these symptoms obliged the patient to enter the hospital. There was no œdema of the limbs, and but moderate palpitation; there was no important previous treatment, and the patient was not strictly confined to her bed.

We now inquire if the disease can here be recognised at the first glance, from the detail of symptoms gathered from the patient. It cannot. Let us enter into an examination of the symptoms. In the first place, can the affection be neuralgia? It has some points of resemblance with this disease, but it differs from it, in many particulars which are sufficiently well marked. First, it is not common for any considerable degree of cough to exist in neuralgia, nor have we in it, a steady, local pain, as in the case described. Another set of symptoms, which establishes the difference between the two affections, is that belonging to the countenance, the medical physiognomy of the case. This is characteristic, not of neuralgia, but of an intense pectoral disturbance.

Satisfying ourselves that it is not neuralgia, we make a general diagnosis of an acute disease of the chest, first, from the severity of the dyspnœa present, established by the spasmodic contraction of the chest and of the nostrils; and, secondly, by the alteration in the colour of the countenance, in the dark blue tinge of the lips and flush of the cheeks. This is not purple enough for the existence of pneumonia, and we, therefore, infer, that our case is, probably, one of bronchitis, pleurisy, or acute phthisis.

Having carried our examination thus far, let us proceed to discriminate between the affections, to which we have reduced the case. This can be done only by physical exploration, without which it is impossible to recognise with absolute certainty the distinctive features of the disease. What, then, are the physical signs, which establish the character of the affection before us? First, we have an abnormal development of one side of the chest, at the lower part, and diminishing gradually in ascending. This, at once, settles satisfactorily the nature of the affection; it is a pleurisy. Had the development been confined to the anterior part of the chest, it might have been emphysema, or, had it been local, pericarditis; but only a pleuritic effusion could have made it what it was. Next, we have immobility of the ribs: in the present case this could result only from pain, or from old adhesions. The history of the case disproves the probability of the existence of old adhesions on the right side; so that from the pain, then, we again deduce the presence of acute pleurisy.

Continuing the examination, we next proceed to percussion, which yielded the following results. There was flatness in the lower posterior half of the side of the thorax, and of the lower third on the anterior part; as well as of the lower half of the axilla. Thus far, we can diagnosticate, with certainty, the existence of pleurisy; the flatness followed the line of gravity, or, in other words, corresponded with the situation of the liquid, which, following the ordinary laws of liquids, accumulated in the bottom of the chest, just as if it had been contained in a common bag.

We continued the examination, by auscultation. Had the disease been pneumonia, we should have had bronchial respiration and a dry crepitus. This was not the case, as the lung was quite permeable to the air, and not a hard, solid mass. There was no rhonchus, for there was no liquid in the bronchial tubes. The physical signs, then, have led us to a certain conclusion, as to the nature of the affection.

The next question, that presents itself, is, as to the primary or consecutive character of the disease. To solve this question we entered upon an examination by auscultation of the other side of the chest; there was, here, no evidence of the existence of tubercles, in any great numbers, but, from the fact of their being some dulness on percussion, there was reason to suspect their presence, though not to determine it, with any certainty. But from the circumstance of the mutability of the situation of the pain, as there had been, you know, a previous attack on the other side of the chest, the existence of tubercles was rendered probable. It is a law of pathology, that, if a pleurisy appear on one side of the chest, and some time afterwards show itself on the other, it, in all likelihood, is dependent on the presence of tubercles. This law of the double pleurisy, as it is called, was discovered by Dr. Louis, and, in most cases, may be relied on with certainty. I infer, then, that, in this case, the pleurisy was probably tubercular.

Besides the suspicion of tubercles as a predisposing cause of pleurisy, the patient is laboring under an undoubted disease of the heart. It began during the acute inflammation of the early part of the winter, when the pericardium was involved at the same time with the pleura. As a consequence of that inflammation, we have hypertrophy and dilatation of the heart.

The treatment of this patient was active; she was bled to sixteen ounces, was cupped, and has since taken tartarized antimony and digitalis. Under these remedies, with rest and restricted diet, she is rapidly improving.

The remedy here used, tartar emetic, is found to answer perfectly well in the management of pleurisy. It increases the sweating, and promotes absorption directly; the digitalis has a similar action through the medium of the kidneys. In pleurisy, when the inflammation is circumscribed, local depletion is the best treatment. This acts very promptly in serous inflammations, although it has but slight influence over the inflammations of parenchymatous structures. In the latter case, relief is afforded, only in proportion to the quantity of blood abstracted, while, in pleurisy, it is in proportion to the nearness of the point of abstraction to the seat of pain. The pain is to be relieved, also, by the application of warmth to the part, by poultices and fomentations. These, however, are not to be withdrawn suddenly, or without the substitution of a thick pad; otherwise, they only do harm, by the alternation of heat and cold, which takes place. The advantages of these local applications cannot be too highly estimated. I often rely more upon them, than upon more powerful remedies, which, if they relieve pain, at the same time diminish the strength.

The treatment of ordinary simple pleurisy is not a very complicated affair. And I would remark, that every case, in which there is no positive evidence of a change in the functions of nutrition, even though there may be strong ground of suspicion of a tuberculous or other chronic disease, is to to be regarded in the treatment as a simple pleurisy. The treatment of Dr. Louis, which I do not, however follow in all cases, consists in small bleedings, combined with the internal administration of tartar emetic, nitre, digitalis, and diuretics. Of sixty cases, that I saw him treat, he lost not one. It may be inferred, that is an effectual treatment for the management of simple pleurisy. I may add, that, in a simple case, if effusion take place, you need not be very uneasy, if it is slow to absorb, provided the case is otherwise proceeding well.

Of the remedies, by which chronic pleurisy is to be managed, (meaning, by chronic pleurisy, cases of more than a month's duration,) I shall not now treat in full, giving you merely a general summary of them. Blisters, which, in acute pleurisy, are not often necessary, and do harm twice for once that they do good, are of signal service in chronic pleurisy, scarcely ever doing mischief, and often effecting a rapid absorption of the liquid effused. They are to be applied not once, but repeatedly. Under their influence, absorption sometimes takes place, with astonishing quickness; indeed, it seems, in a few instances, as if the fluid was directly poured out from the interior of the chest, to the blistered surface, by a sort of endosmosis. Mercurials, in small doses, are not much used by the French, either alone, or in combination with squills and digitalis. But, in cases approaching in character to hydrothorax, great advantage will be derived from a treatment with calomel, squills, and digitalis. In addition to treatment by medicines, travelling, a sea voyage, distractions, a simple change of place, will be of much service. The importance of travelling is greatest in those cases in which we fear the complication of a tuberculous diathesis. Whether there be already formed tubercles in the lung, or merely the constitutional tendency to these affections, I am quite sure that by this means and by attention to other hygienic circumstances, patients are often preserved from a threatened consumption.

In addition to the case illustrative of one of the most simple serous inflammations, I shall say a few words respecting another case, in which pleurisy occurred, as a well marked complication. The case was one of pneumonia, consecutive upon tubercles, the existence of which was known by unequivocal signs at the upper part of the lung. There was something, however, engrafted on the pneumonia. This was pleurisy, and was detected by a sign which often occurs in the later stage of the affection, and is then pathognomonic, the bruit de frottement, a sound caused by the friction of the surfaces of the pleura, lined with false membrane, upon each other. It resembles the sound produced by the rubbing of leather or India rubber, and is the same grating sound that was heard over the heart of the man Robb, but in the present case it is produced by respiration, and is synchronous with it. It is, also, fugitive in its character, and disappears, when the membranes become consolidated.

I shall conclude with one other case, which terminated fatally a few days since, demonstrative of one of the causes of serous inflammations, the details and phenomena of which will serve as a key for future investigations. It was a case of chronic tubercular peritonitis. My reasons for this diagnosis were based upon, first, the conformation of the abdomen, which was irregularly distended with gas, upon the existence of lancinating, griping pains, or alternations of costiveness and looseness of the bowels, and upon the pain caused by motion, or the distension consequent upon eating; there was, besides, nausea and vomiting. The peritonitis occurred here without any obvious cause, and was, therefore, not primary. For there is a law of the economy that chronic peritonitis is nearly always, particularly in young persons, dependent on the presence of tubercles. In addition to this general law of pathology, the great alteration in the nutritious functions made the diagnosis of the existence of tubercles much more certain. It was, at first, doubtful, from the large distension of the abdomen from serum; but the water here was soon absorbed, and there was no recurrence of ascites. There was evidence also of tubercles in the lungs, although not very decidedly marked. Had there been physical signs of phthisis the case would have been still positive, although we found a sufficient number of signs for the diagnosis.

Most commonly, tubercles appear in the lungs of adults, before they are deposited in other parts of the body; but, in this instance, the application of this general law failed. The patient, some days before his death, was seized with sudden prostration, under which he rapidly sunk, and with some increase of the abdominal pain.

After death, the following appearances were discovered. There was effusion of serum and pus into the abdomen; in the upper portion there was merely serum and lymph, and, in the lower, the intestines were agglutinated by false membranes perfectly organized, not vascular, but there was red injection in the upper part from a more recent inflammation. The cause of these changes was perforation of the intestine, from tuberculous ulceration of the glands of Peyer, two of which had ulcerated, through all the coats of the intestine, into the peritoneum. The pathology of these perforations is the following:—A tubercular follicle in the intestine, enlarges and softens, and is discharged into the caliber of the gut. The ulcer left does not heal, and, passing into the chronic state, advances towards the serous covering of the intestine, which is sometimes destroyed. The peritoneal inflammation is only an attempt of nature to preserve life by preventing the discharge of fœcal matter into the peritoneum. It fails, because the mischief done is too considerable to admit of reparation.

In the lungs, the only evidence of the presence of tubercles, were half-a-dozen grey granulations, that could be felt, but scarcely seen; while, in the peritoneum as well as in the intestines, they existed to such an extent as to cause disorganization. This disease is unusually rife among negroes; indeed, it is sometimes called consumption of the negroes, in the southern parts of our country. It rarely attacks adult males, more commonly females, and is very prevalent with children, in whom it forms one of the diseases known as tabes mesenterica, although the mesenteric glands are not invariably affected.

To recapitulate my remarks of to-day:—Serous inflammations may appear as primary and secondary. When primary, they are not dangerous; but they are so, when secondary, because complicated with some previous lesion, and occurring in exhausted subjects. They are to be treated, in both instances, on pretty much the same principles, by depletion, and acting on the skin, with alteratives in the more chronic stages. If excessive pain exist narcotics may be used to relieve it, with the topical applications you may see every day employed at the hospital.

For the proper study of tuberculous diseases, gentlemen, a knowledge of the pathology of serous membranes is indispensable. Previous, then, to entering upon the examination of the former affections, I have introduced the subject of serous inflammations, to facilitate our future investigations. The study of tuberculous diseases is not, as you have also seen, to be confined to the chest, but to be extended to all the organs of the body, as you will more fully learn at a later period.

The tuberculous affection of the abdomen, which has been under consideration to-day, though not the most common form of the disease, in our latitudes, is one of the most prevalent in southern climates, and is on that account the more interesting to many of you.

---

### TUBERCULAR MENINGITIS.

*Tuesday, May 8th.*—I shall continue to-day, gentlemen, said Dr. Gerhard, the subject of inflammations of the serous membranes, and take up the consideration of a case, which came under your notice at the hospital, a day or two since, and presented an example of inflammation of the serous membrane investing the brain. We may the more properly enter upon the subject at this time, as it will facilitate a future examination of the diseases of the substance of the brain.

The patient, of whom I have spoken, died in the ward, No. 3, and was not under my immediate care. He was a carpenter by trade, and had suffered severely in early life from scrofulous affections; both his feet had been ulcerated from this cause, some time previous to his admission. He also laboured under disease of the heart. He entered the hospital for hydrothorax, the cavities of both pleuræ being filled with water, and he suffered under an extreme and distressing dyspnœa. He was relieved from these symptoms by salivation, combined with the use of digitalis and squill. He got rid of his shortness of breath, and was able to work in the out-wards of the establishment, where he continued, until the breaking out of the epidemic of measles, with which disease he was taken on the second of April. He suffered considerably from the measles, but gradually became convalescent, till, on the twenty-eighth of April, he offered some symptoms of a cerebral affection, that is, unusual dulness, stupor, and oppression. On the first of May, the cerebral symptoms became so well marked, that they were recognised as those of meningitis, by the physician in attendance. At that date, he was in the following condition. For two days previously he had manifested great restlessness, with occasional incoherence and hallucinations. Skin warm; pulse full and strong, the bis feriens character, attending his convalescence from measles, having ceased from the twenty-ninth of April. This bis feriens character, a sign of convalescence from the measles, and which was well marked in this case, ceased, you see, the moment the man was taken with the new disease of the brain. The thirtieth, he was bled ℥ix; the crassamentum of the blood was tolerably firm, and it was neither cupped nor sizy, about one half of it being serum. The man at this time answered questions slowly. The conjunctiva was slightly injected. The tongue pale, moist, slightly furred. The pupils insensible to light, although he still recognised objects. No cephalalgia. The abdomen resonant; not painful on pressure, except in the hypogastric and pubic regions, where it was also distended and flat on percussion. Percussion of the left side of the chest resonant, except in the præcordial region; respiration pure. Percussion of the right side resonant, but less so than the left. Impulse of the heart strong; the first sound prolonged, attended with strong bellows murmur in the neighborhood of the nipple, the same character of the first sound observed between the second and third ribs; the second sound roughened, and heard over an unusually large extent of the right side. A purgative of salts and senna was prescribed, and a blister to the nape of the neck, dressed with mercurial ointment.

On the third, the countenance was rather less dull than on the first, and he answered questions better; had been delirious the night before. There was some grinding of the teeth. The skin was moderately warm and hot. Pulse ninety per minute, and much smaller. Conjunctiva much redder; a discharge of a small quantity of yellowish matter from the right eye. In the afternoon, there was some strabismus and increased stupor. An injection of oil of turpentine and castor oil was administered, and cold applied to the head.

On the fourth, there was very great stupor; the eyes were closed, and the patient could not be roused to answer questions. The head was turned to the right side; the right eye inflamed as before; the pupil of the left eye smaller than yesterday. Pulse about eighty-five, irregular and moderately strong. Bowels opened three times by the injection. Abdomen supple, and not distended. Cold to the head continued; calomel, followed by senna and salts. The same day, the man died.

The symptoms here, you perceive, were not those, which denote active, violent inflammation, but were simply dulness of the intellect, stupor, with grinding of the teeth, &c.

The treatment, you have noted, was commenced by a large bleeding, which was followed by purging, and an attempt to mercurialize the patient, which latter failed, from the short time that elapsed between the administration of the remedies and the man's death.

The following appearances were found in the brain, twenty-four hours after death.

Marked adhesion between the dura mater and the membrane beneath. The vessels of the dura mater were more congested than usual. In taking the brain from the cranium, about two ounces of fluid escaped. The large vessels of the pia mater were much conjested; the capillary vessels, of a bright red tint,—inosculating. In the middle part of the right side, the convolutions were flattened; on this side, the injection of the pia mater extended to that portion dipping into the convolutions, and it adhered strongly to the cerebral substance. The injection and adhesions were less marked towards the posterior portion. At the anterior extremity, the arachnoid membrane was opaque; the injection and adhesion somewhat less than at the middle. On the left side, this bright injection occupied the middle half, and was confined almost exclusively to the small arterial vessels. Pia mater less adherent, than in the right side. Arachnoid slightly opaque, throughout the whole extent, presenting a few minute granulations, near the parietal protuberance. The cortical substance on both sides was of a rosy tint, a little brighter on the left than on the right. That portion of the arachnoid, covering the fissure between the hemispheres, and at the summit of the brain, was slightly roughened. Corpus callosum softened. Fornix and septum lucidum pulpy. The right ventricle was larger than the left; the quantity of serum contained, not known. The thalami and corpora striata were pale. At the base of the brain, the colour was, in a great measure lost, from the commencement of decomposition; but, in the whole anterior hemisphere, injection of the small vessels was manifest. There were small adhesions between the anterior lobes of the brain. Fissure of Sylvius, on the left side, strongly adherent, by a solid deposit around the vessels, part of which, in the form of granulations, was still distinct. On the right side, the same thickening occurred around the vessels, but the newly formed matter was less abundant than on the left side; it still presented granulations, less in size than a pin's head. The arachnoid was opaque and extremely thickened; the thickening of this membrane extended backwards over the chiasm of the optic nerves, which it slightly invested. Towards the cerebellum, the thickening of the membranes became more marked at the upper portion, at the point of junction with the cerebrum; the double secretion was there distinct, consisting, in part, of minute granulations, beneath the membrane, and in its thickness, and, in part, of a thick, opaque, hard substance, filling up the space between them. The cerebellum was firm, like the rest of the brain.

There were no tubercles in the lungs, or the viscera of the abdomen. The state of the lymphatic glands was unfortunately not noted by the gentlemen who made the examination. From the former scrofulous disease, these glands were probably tubercular.

It is to be inferred, then, that the disease of the brain was here of an inflammatory character, from the injection and thickening of the arachnoid membrane. It was evidently of the tuberculous variety, from the granulations which were found scattered beneath the arachnoid—it was a case of tubercular meningitis. The bright injection of the arachnoid, which is limited to the smaller vessels, is a very good diagnostic sign of the disease; had it been observed in the larger vessels merely, I should have regarded it as a simple conjestion. In the present instance we have, then, only the alteration in the membranes of the brain, to account for the cerebral symptoms, as the substance of the organ is not at all affected.

This subject of tubercular meningitis, gentlemen, is one that will present itself frequently to your notice, as it is a disease very common with children, and by no means rare in adults. It is generally slow and insidious in its progress, and requires a very careful examination to distinguish it, particularly in its early stage. I have taken, for the subject of some general remarks to-day on this disease, a case, in which we have had the pathological phenomena very clearly presented to us, and in which the indications, previous to death, were sufficient for a correct diagnosis of the affection. This case, I may remark, exemplifies the occasional effect of measles, in giving rise to the development of tubercles, to which I alluded at my last lecture.

This individual, we learned, had had an attack of brain fever, (so termed by his mother,) many years ago, by which his mind was at the time considerably affected. This was probably a scrofulous inflammation of the same character, as that which finally carried him off. Children may recover from these tubercular cerebral affections, and, at some subsequent period of their lives, present the same symptoms in a more marked manner, from a new secretion, or, as it were, a second crop of tubercles in the membranes of the brain. So, patients may partially and temporarily recover from pulmonary phthisis, as was shown to you in a late autopsy, where co-existing with the cavities which immediately preceded the death of the patient, were distinct traces of the operation of a former cure, in the hardened cicatrices, which we found in various parts of the lung. The man, whose case we are considering, had probably recovered from an attack of tubercular meningitis, early in life, and he might have remained well, had not the occurrence of measles awakened the slumbering tubercular disposition, and caused a fresh development of the affection, which now proved fatal.

This subject of tubercular meningitis, I investigated very fully some years ago, at the Children's Hospital at Paris, and obtained some important results as to the nature and causes of the affection. The first point of inquiry, upon entering on the subject, is, have we any evidence of the existence of tubercles, elsewhere than in the brain and its membranes, in this affection? In the children who died from this form of inflammation of the brain, I found tubercles in the bronchial glands or other organs of the body, as well as in the substance and membranes of the brain, where they were found from the size of a pin's head to that of a large pea.

There was but one evident exception to this rule, out of thirty cases, which were analyzed in a paper, which I published in the American Journal, in 1834. In the exceptional case, there were tuberculous granulations in the membranes of the brain, but not in other viscera. The coincidence of tubercles in various parts besides the brain conclusively proves, that a general tuberculous diathesis existed in these subjects, for in no other class of acute disease, does the same rule obtain.

Having determined the point of the general tubercular nature of the disease, the next matter to be investigated, is, the causes on which depends the development of the affection. Unquestionably, the scrofulous diathesis is the strongest predisposing cause of this affection, using the word scrofulous as significative alike of the tubercular and strumous temperament. In almost all the cases in which the cerebral affection occurs in adults, a scrofulous disease has previously existed, and perhaps been cured, in some other part of the body, as the lower extremities, the glands of the neck, the lungs, and elsewhere. As to the exciting causes of this disease, they escaped us almost entirely; in the majority of cases, at the Enfans Malades, we could detect no antecedent fact, which could at all account for the development of the tubercular disease of the brain. The measles, however, in the case under notice, is to be looked upon as the accidental cause of the development of the disease; and I may make the general remark, that, whenever, in a scrofulous child, you have an acute disease accompanied with fever, you may look for the development of inflammation of the brain, and are to watch your case with exceeding care.

The prognosis, in tubercular meningitis, must, generally, be more or less unfortunate, particularly in hospital practice. This deduction I base upon the observations, made by myself and my friend Dr. Rufz, at the Children's Hospital at Paris, where, for one or two that got well, forty died. Indeed, Charpentier, who observed ten years ago in the same hospital, went so far as to say that he never saw one case recover. Yet, in private practice you will find the results much more favorable. In the hospital at Paris, the children were badly fed, confined in close rooms, and the treatment prescribed was not so minutely carried into execution as in private practice. I have not seen many children with this complaint since my return to Philadelphia, but those cases which I have seen, were generally but not always fatal. A striking instance occurred in the child of one of our nurses; she was a girl of four or five years of age, and recovered, entirely, but a second attack came on, a year or more after the first, and proved fatal.

The adults, who were taken with tuberculous meningitis, were all labouring under phthisis pulmonalis, which complication contributed not a little to the fatal termination of the affection. These cases, were, of course, not fair subjects for estimating the powers of treatment. In many cases, also, of this disease, the existence of tubercles in the lungs may not be ascertained during life, although they are found after death. This was the case with a negro, whom we examined some years since at the Pennsylvania Hospital. (See *American Journal*, 1836.) During life, he had neither cough nor expectoration, but we found, after death, numerous miliary tubercles in the lungs, as well as in the brain; in other words, the man laboured under general acute tubercular disease, which from the similarity in the size and appearance of the granulations, commenced nearly at the same time in the lungs and brain, but had not attained a large size in either organ. Generally, we meet with the disease in adults, under circumstances that preclude the hope of a cure; but, in children you may entertain a fair hope of success, if you see the case early; if it have advanced so far as the second stage of the disease, you have but a mere chance of saving the child from speedy death.

The symptoms differ in children and in adults. In adults, the disease appears in patients actually labouring under phthisis, or of a decidedly strumous diathesis; while it often shows itself in children, who are in the enjoyment of tolerably good health, notwithstanding some latent tendency to scrofulous diseases. Whenever in children, the symptoms which I shall describe as characteristic of the forming stage of the disease, present themselves, the physician should put himself upon the watch, though they are not to be looked upon as invariably indicative of the result in question. Tubercular meningitis may begin in two ways. First, it may come on abruptly, as ordinary acute meningitis, with vomiting, chill, and fever. The cerebral symptoms may appear, however, without even the prelude of vomiting; in adults this symptom is commonly wanting. When its approach is more gradual, the following order of phenomena is observed:—

For the first few days, the child merely evinces unusual restlessness and irritability, showing signs of the excitement of the brain. He avoids light and sound, from the extreme sensibility of the eyes and ears. We have also a change in the intelligence, if the child be old enough to permit such change to be noticed. First, it is simply excited; the child is more lively and acute, and more attentive to external objects, than before. Afterwards the countenance becomes changed; the cheeks are flushed, the eyes unusually bright, and a well-marked frown and wrinkle are to be noticed upon the forehead. This is one of the most important signs of the early stage of the disease; and at this period, it is essential to recognise all the symptoms, and this peculiar expression you may consider characteristic. This, together with the bright red flush upon the cheek, the nurses in the Children's Hospital used to look upon as an unfailing mark of the approach of the disease. The decubitus is at this time but slightly altered. But we often meet with some secondary symptoms, which, although they are not always present, are of some moment; these are nausea or vomiting, constipation, and fever which is at first of a mild character.

We now pass to the second stage, comprising the symptoms which were first observed in the man who has just died; those of the forming stage were lost in the decline of the measles. These symptoms are delirium, which cannot of course be very accurately observed in children, particularly, if they are very young. But some signs of it may be generally detected, especially at night, in the quick answers and altered manner of the child. This delirium differs from that of ordinary acute meningitis, in which the patient is violent, noisy, and loquacious. Here there is mere dulness and stupor somewhat similar to the delirium of typhus fever; the child is not very violent, makes no efforts to walk about or to do mischief, but remains in a state of dull muttering.

I was impressed with this peculiarity of the delirium of tubercular meningitis, in two cases which came under my observation at the Pennsylvania Hospital, three years since. In one, of so moderate a character was this delirium, that the patient was admitted for simple insanity. The only other symptoms that attracted attention, upon his admission, were a peculiar hobble and limp in his gait. We found the traces of several scrofulous disorders, which had been cured, and the man had also a slight cough of which he complained for two years past. The patient was constipated before his entrance, and shortly afterwards, vomiting ensued, and then the cerebral symptoms became more decided. The paralysis occurred very early, from the coincidence of softening and inflammation of the substance of the brain, with that of the membranes. At first, in fact, I thought it was paralysis, from mere softening of the brain. Afterwards, I began to doubt, and regarded the case as one of tuberculous meningitis: finally, the autopsy cleared up all obscurity. The paralysis was found to be dependent on softening of the brain, and the delirium arose from tubercular meningitis with effusion of lymph at the base. This complication of lesions necessarily gave rise to the intermixture of the symptoms of meningeal with those of cerebral inflammation. In practice this coincidence is by no means rare, and it is not often difficult to detect it. The seat of the disease was here the same as in the case of the man Crane; the deposit of tubercles was along the blood vessels, following the ordinary law which regulates the secretion of tubercular matter.

Besides the delirium of the second stage, we meet with alteration of the senses, as in the case of Crane. The pupils are generally dilated, moderately and gradually; in some cases, they are contracted, but, as was observed by Dr. Stewardson in the present case, it is difficult to tell when they are permanently contracted, being at one time contracted, and at another dilated. These alterations in the pupils are most important signs, particularly when accompanied by the muttering delirium.

Lesions of motility next present themselves. These consist at first principally in subsultus or even spasms, as in typhus fever; indeed I have sometimes hesitated for a little while in my diagnosis, between the two affections. Paralysis is by no means a necessary symptom in the second stage of the disorder. But we have now the beginning of another symptom, rigidity. In the case of a man now in the wards, this stiffness could be detected only by careful examination of the elbow, but it may be usually very early ascertained with proper caution. This rigidity differs from contraction, which is a more advanced degree of it, and is more rarely met with in this form of meningitis. Rigidity is not here confined to one side of the body, as in apoplexy and softening of the brain. The tubercles are secreted, on both sides of the base of the brain, and, hence, the symptoms of disease of the membranes are rarely confined to one-half the body, while those of the cerebral substance are rarely extended beyond it.

These are the chief cerebral symptoms of the second stage of the affection. We now pass to another set, those of the digestive organs. Vomiting is one of the most constant symptoms of tubercular meningitis, in children, but it rarely continues beyond the first stage. Another peculiar and important symptom is constipation. In the second of the two patients in the Pennsylvania Hospital, to whom I have alluded, the case was at first looked upon by his physician as one of simple constipation; and the true nature of the complaint was suspected only when it was found that this symptom did not yield to purging. This symptom gives us a valuable therapeutic indication, in the treatment of the affection—the propriety of purging. The appetite is generally lost from the beginning of the affection. The thirst is in proportion to the degree of fever present. The state of the pulse may be learned from the case of Crane. In him, the bis feriens pulse, of 40 and 66 per minute, existing during the convalescence of measles, rose at once to 90, and continued at this point till the third stage, when it sunk again to 85. It was therefore simply febrile in the second stage, and irregular in the first and third. It is rarely slow, and slowness may be looked upon as always a good symptom.

The other symptoms are less significant in their character, and I would merely refer you to the memoirs which I published in the American Journal, in the years 1834, '35.

In the third stage, or after effusion of serum, pus, or lymph has taken place, the ordinary termination of serous inflammations, to which I called your attention in my last lecture, we have a subsidence of the acute febrile disturbance, the pulse is often preternaturally slow, and partial paralysis presents itself, from the pressure of the effusion, which is not necessarily confined to one side of the body, and is slow and gradual in its advances.

I have given you merely a slight sketch of the pathological anatomy of this affection, as I do not, in this course, intend to dwell, at any great length, upon this subject. The treatment of tubercular meningitis, to the consideration of which we now pass, involves many important questions. It must vary, according to the severity of the actual symptoms, and the circumstance of the existence of a previous tubercular disease. If the patient is in the third stage of phthisis pulmonalis, you can of course do little or nothing. If this be not the case, however, you may, I think, do much. The case must be at first treated as one of simple meningitis. Your object should be to get rid of the acute inflammation of the brain, the existence of which increases the disposition to tubercular secretion, and may at once kill the patient. You must not, however, deplete to the same extent that would be advisable, if there were no tubercular disposition. You are to steer a middle course. My plan is to resort to blood letting, general and local, unless the development of tubercles be very strongly marked. I have recourse to general blood letting, once, and once only, even in adults. It is an old remark of writers, that inflammations of the membranes of the brain generally bear extensive depletion worse than those of other organs, but always tolerate well the local abstraction of blood. Local bleeding is to be directed, so long as the patient can bear it, that is to say, until he becomes pale, and the flush is gone, whether the other symptoms abate or not. After depletion, I was formerly in the habit of placing blisters to the back of the neck. I am now in the practice of applying them behind the ears. The discharge can here be kept up longer, and will act more steadily, and the sore can be better dressed; the patient may be mercurialized by dressing these blisters with mercurial ointment. The discharge by the blisters I keep up, until the patient is perfectly well. Another remedy is counter-irritation elsewhere than in the head. The feet are apt to be cold; they are to be plunged into hot water, from time to time, to be clothed with flannel, and rubbed occasionally with cayenne pepper. But you are to abstain from blistering; it only serves to create fever, and is generally mischievous. Sinapisms may be used, but the surface is not to be vesicated.

The next remedy to be employed is purging. If the patient be strong and robust, it answers a very good purpose, and in a few rare cases at once relieves him. But in children, if relief be not afforded by one or two purgative doses, it is proper to be cautious as to their employment. With children I begin with a mercurial purge, from four to eight grains of calomel, to be followed by a saline purgative. Mercurials are used by the French merely for the purpose of purging; of course they do not salivate, and, when persisted in, do no good. If the acute stage of the disease have abated, you must commence with mercurial dressings and frictions to the abdomen. These are of most service in the sub-acute variety of the disease.

I have now detailed you the ordinary practice, to be observed in the management of tubercular meningitis. To one or two points, your attention is to be particularly directed. You must carefully watch the moment, when it is proper to stop blood-letting, and immediately after commence the introduction of mercury into the system.

After the third stage of the disease is established, and paralysis makes its appearance, treatment can do no good. The affection is fatal, because the functions of the brain cannot be interfered with, with impunity.

Tubercular serous inflammations are not elsewhere so fatal, as when they occur in the membranes of the brain. When secondary peritonitis and pleurisy destroy life, they usually arise from perforation of the glands of Peyer, and from perforation of the lungs.

The tuberculous inflammations of these membranes, however, assume a much higher importance from their disposition to return, and even to attack other portions of the body. Besides, they certainly favour the development of tubercles, in cases in which the patient may have previously presented merely the diathesis which precedes this morbid deposit. For a more complete account of this connection, I must refer you to my lectures on pathological anatomy.

The symptoms and treatment of tuberculous meningitis you will find detailed in the memoirs which I published in the American Journal, in the years 1834—5, as well as in the paper of my friend, Dr. Rufz.

